# Aberrant immunophenotypic expressions in childhood acute leukemias: A tertiary care hospital experience

**DOI:** 10.12669/pjms.41.3.9717

**Published:** 2025-03

**Authors:** Nazish Saqlain, Sidra Hareem, Fatima Batool, Khalid Mahmood

**Affiliations:** 1 Nazish Saqlain, FCPS (Hematology) Associate Professor of Pathology, Department of Hematology & Transfusion Medicine, UCHS, The Children’s Hospital Lahore, Lahore - Pakistan; 2 Sidra Hareem, FCPS (Hematology) Assistant Professor of Hematology, Department of Hematology & Transfusion Medicine, UCHS, The Children’s Hospital Lahore, Lahore - Pakistan; 3 Fatima Batool, B.Sc. MLT Medical Laboratory Technologist, Department of Hematology & Transfusion Medicine, UCHS, The Children’s Hospital Lahore, Lahore - Pakistan; 4 Khalid Mahmood, B.Sc. MLT Senior Medical Laboratory Technologist, Department of Hematology & Transfusion Medicine, UCHS, The Children’s Hospital Lahore, Lahore - Pakistan

**Keywords:** Acute leukemia, Children, Immunophenotypic expression, Aberrant markers, Minimal Residual Disease

## Abstract

**Background & Objective:**

Pediatric acute leukemias can present with aberrant immunophenotypes characterized by a different pattern of antigen expression on malignant cells, unlike the process of usual hematopoietic maturation. The objective of this study was to determine the aberrant immunophenotype expressions in newly diagnosed pediatric acute lymphoblastic and acute myeloid leukemias.

**Methods:**

This cross-sectional study was carried out at University of Child Health Sciences, The Children’s Hospital, Lahore, from October 2022 to December 2022 after IRB approval. After taking informed consent from parents/guardians, 290 children diagnosed with acute leukemia were included in the study. Peripheral blood or bone marrow samples in EDTA vial were used for flowcytometric analysis by using BD FACS Canto II flow-cytometer. The data was collected on a pre-designed proforma and analyzed by using IBM-SPSS V-23.

**Result:**

Among 290 cases, Acute Lymphoblastic Leukemia (ALL) constituted 221(76.2%) cases and 69(23.7%) were Acute Myeloid Leukemia (AML). Out of the total, the aberrant antigens were present in 32(11%) patients with 40 total events (18.1% immunophenotype aberrancy rate). The most common aberrant antigens were reported in B-ALL and the most common aberrant expression was of CD13 (62.5%). In AML, the most common aberrant antigen seen was CD19 (55.6%) and in T-ALL the most common were CD117 and HLA-DR (26.6%).

**Conclusion:**

The most aberrant immunophenotypic markers were seen mostly in B-ALL pediatric cases followed by AML and T-ALL. Such abnormal expressions should be kept in mind while diagnosing the children with acute leukemia as they may affect the prognosis.

## INTRODUCTION

Leukemia is characterized by an unchecked proliferation of immature cells in the hematopoietic system replacing the normal elements.^1^ Leukemias are classified as acute and chronic depending on the time duration of symptoms and as lymphoid, myeloid and mixed lineage phenotype depending on the ancestry of malignant cells.^2^ Acute leukemias generally have an aggressive course if left untreated. Acquired genetic damage causes cellular proliferation to increase, apoptosis to decrease and differentiation to stop. Together these processes lead to an increase of blast cells. Acute Lymphoblastic Leukemia (ALL) is caused by the accumulation of lymphoblasts in the bone marrow, blood and extramedullary sites. Acute Myeloid Leukemia (AML) is defined as an increase in the number of myeloid blasts in bone marrow or blood at clinical presentation.^3^

Aberrant phenotype in acute leukemia is a phenomenon of abnormal expression or loss of expression of cell specific lineage marker not associated with specific cell type due to their abnormal genetic program.^4^ Blasts cells in some cases of B- ALL may lack certain B lymphocyte antigens and possess T-cell and/or myeloid antigens. Similarly, blasts from the cases of T-ALL may possess some B-cell and/or myeloid antigen. Some lymphoid lineage associated antigen show their expression on AML blasts. This phenomenon is called aberrant expression of CD (Cluster of Differentiation) makers, determined by flowcytometry. Aberrant antigen expression is of a prognostic value and may have an adverse effect on clinical response, remission rate and overall survival in patients with acute leukemia.^5^,^6^

Our study aimed to evaluate the aberrant immunophenotypic expressions in pediatric acute leukemias. Currently, there is limited local data regarding the characteristics of different types of acute leukemias in children. At present, well-organized cancer registry programs in the country to keep a track of these malignancies are limited. This is of utmost importance to know the antigen profile and its effects on long term survival in childhood acute leukemias in our population. The objective of the study was to determine the prevalence of aberrant immunophenotype expressions in newly diagnosed pediatric acute lymphoblastic and acute myeloid leukemias.

**Fig.1:**
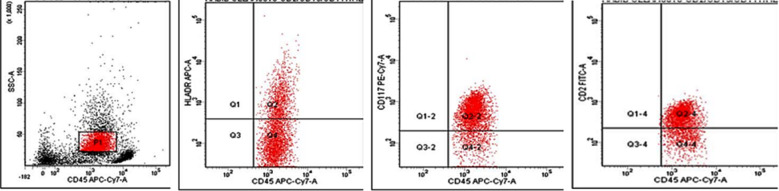
Case of B-ALL with aberrant expression of CD13.

**Fig.2:**
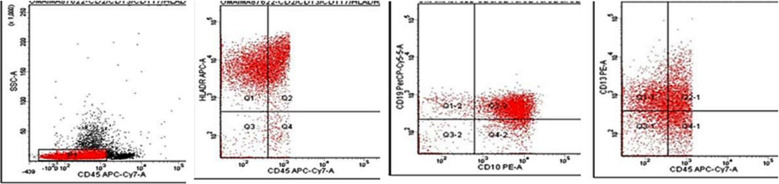
Acute myeloid leukemia with aberrant expression of CD2.

## METHODS

It was a cross-sectional study, conducted at The University of Child Health Sciences, Lahore from October 2022 to December 2022. In this study, 290 children (1-16 years old) diagnosed with acute leukemia were included after taking informed consent from parents/guardians. The confidentiality was maintained at each level of the response in the study.

### Ethical Approval:

The study was approved by the Institutional Review Board (1210/SAHS dated 11/08/2022).

Diagnosis of Acute Leukemia (AL) was made on the basis of acute clinical presentation and presence of blasts in peripheral blood or bone marrow confirmed by morphological examination of Giemsa-stained peripheral blood and/or bone marrow aspiration smears, cytochemistry, and flowcytometric immunophenotyping. Flowcytometric analysis was done with the monoclonal antibody panel of acute leukemia using minimum of 1ml EDTA anticoagulated fresh Peripheral Blood (PB) or Bone marrow (BM) aspirate sample by standard stain-lyse-wash method. Department standard operating procedures (SOPs) and manufacturer’s guidelines were used to perform flowcytometric analysis by using BD FACS Canto II flow-cytometer. The threshold for positivity was taken as expression in ≥ 20% blasts cells for surface antigens and ≥ 10% blast cell for cytoplasmic antigens. Results were obtained by gating the blasts cells with SSC versus CD45. The primary panel consisted of CD2, cCD3, CD5, CD10, CD19, cCD79a, CD38, CD13, CD117, MPO, HLA-DR, CD34, Tdt, CD14 and CD11c. The additional markers included CD7, CD33, CD64, CD41a, CD61, CD4, CD8, surface CD3, CD20 and Glycophorin-A. Before beginning the procedure, all quality control measures were carried out. The data was collected on a pre-designed proforma. The abstract was presented as poster in International Society of Lab hematology (ISLH) 2023 conference.^7^

### Data analysis:

Statistical analysis was done using IBM SPSS version 23. Continuous variables are expressed as Mean ± SD and categorical variables are expressed in the form of frequency and percentages.

## RESULTS

Of 290 patients diagnosed with acute leukemia, 170 were males and 120 were females with male to female ratio of 1.4:1. Flow cytometry analysis was done using bone marrow aspirate sample in 178(61.3%) patients and peripheral blood sample in 112(38.6%) patients. The range of age was between 1-16 years.

Out of the total cases, 221(76.2%) were reported as ALL and 69(23.7%) were reported as AML. ALL was further classified into T-ALL which were 46(15.8%) cases and B-ALL cases were 175(60.3%). The aberrant antigens were present in 32(11%) patients with 40 total events (18.1% immunopheno type aberrancy rate).

Out of the total T-ALL cases, 8(17.3%) exhibited aberrant markers with five cases showing more than one aberrant expression. The total 15 events (32.6%) of occurrence of aberrant antigen markers were seen in T-ALL including CD10 (20%), CD13 (13.3%), CD33 (6.6%), CD79a (6.6%), CD117 and HLA-DR (26.6% each). The mean age of the patients showing aberrant antigenic expression in T-ALL was 8.8 ± 4.3 (Mean ± SD). The common aberrant antigens found in these cases were CD117 and HLA-DR and the less common ones were CD79a and CD33 which were present in a single case. The five cases with multiple immune phenotypic aberrancies presented with combinations of CD79a and CD10, CD117 and HLA-DR, CD13, CD117 and HLA-DR, CD13 and CD117 and CD33, CD117 and HLA-DR. Table-I

**Table-I:** Aberrant antigen expression in T-ALL.

Aberrant antigen	Aberrant markers expressions (n)	Frequency (%)
CD10	3/15	20
CD13	2/15	13.3
CD33	1/15	6.6
CD117	4/15	26.6
CD79a	1/15	6.6
HLA-DR	4/15	26.6

Among B-ALL cases, 15(8.57%) had aberrant antigens expression and the total aberrancies events were 16 (9.1%). The mean age of the patients showing aberrant antigenic expression in B-ALL was 7.9 ± 4 years (Mean ± SD). One of them presented with more than one aberrant antigen that is CD13 and CD117, while the other 14 cases presented with single aberrant markers which included, CD2 (12.5%), CD5 (18.75%), CD117 (6.25%) and CD13 (62.5%). Table-II

**Table-II:** Aberrant antigen expression in B-ALL.

Aberrant antigen	Aberrant markers expressions (n)	Frequency (%)
CD2	2/16	12.5
CD5	3/16	18.75
CD13	10/16	62.5
CD117	1/16	6.25

Among AML cases, 9(13%) cases exhibited single aberrant antigenic expression including CD2 (11.1%), CD7 (11.1%), CD19 (55.6%) and CD79a (22.2%) and mean age was reported as 8.2± 4.1 years. According to French–American–British (FAB) classification system AML was further classified into M0-M7 categories. Aberrant CD7 and cCD79a expression was present in two cases of acute myeloid leukemia without maturation (AML-M1). In Acute Myeloid leukemia with granulocytic maturation (AML-M2) patients, CD19 was reported in five cases and CD79a in one case. A single case of Acute Promyelocytic Leukemia (APL, AML-M3) showed CD2 aberrant marker. Table-III

**Table-III:** Aberrant antigen expression in AML.

Aberrant antigen	Aberrant markers expressions (n)	AML Type	Frequency (%)
CD2	1/9	APL (AML-M3)	11.1
CD7	1/9	AML- M1	11.1
CD19	5/9	AML-M2	55.6
CD79a	2/9	AML-M2 and AML-M1	22.2

There were some aberrant markers which were present in more than one type of acute leukemia such that CD2 was present in B-ALL as well as in AML, CD79a was present in T-ALL as well as in AML and CD13 was present in both B-ALL and T-ALL.

## DISCUSSION

Detection of aberrant phenotypes is of clinical importance not only for accurate diagnosis of acute leukemia but also for prognostic purposes. Among acute leukemia, childhood acute lymphoblastic leukemia (ALL) is more common (80%) than acute myeloid leukemia (20%) in contrast to adults where AML is more prevalent.^8^,^9^ We performed this study to categorize acute leukemias in children by cytomorphology and to evaluate the incidence of aberrant phenotypes by flowcytometry. There is a variable expression of aberrant antigens in AL based on a range of markers studied, the total number of samples and aberrancy criteria.

Our study showed that there was male predominance of patients diagnosed with acute leukemia. This fact may be seen because the males have greater exposure to environment than females. Kulkarni KP et al have reported similar findings.^10^ The age group of 4-12 years diagnosed with AL showed more aberrant expressions. In order of prevalence, distribution of cases were of B-ALL, AML and then T-ALL. Noronha et al and Supriyadi et al have reported comparable findings.^11^,^12^ Among AL patients, the aberrant antigenic expression was highest among B-ALL cases in our study. The results are concordant with studies done by Sivakumar M et al and Jalal SD et al.^13^,^14^ While another study reported that there is no significant difference between percentages of ALL versus AML in this regard.^15^

The overall immunophenotype markers aberrancy rate was found as 14% which is lower as compared to one reported by Al-Badran et al in Childhood ALL.^6^ This finding might have been obtained due to limited number of immunophenotypic markers studied, particularly in B-ALL cases. The reported incidence of aberrant antigens ranges from 25 to 55%.^6^,^15^ Among T-ALL cases, we found CD117 and HLA-DR as the most common myeloid aberrant markers and CD10 as B-cell aberrant expression. Gupta M. et al have also mentioned CD 117 aberrant expression in T-ALL cases.^16^ However, Mazher N. et al reported CD13 and CD33 as most frequent aberrant antigens in T-ALL.^17^

We found around 9% aberrant expressions in B-ALL cases with CD13, the most prevalent one. The previous studies have also described CD13 as the most common aberrant myeloid antigen in B-ALL.^5^,^15^,^18^ CD33 is the second most frequent aberrant myeloid antigen reported. In our study, CD33 was not performed for all cases of B-ALL so the percentage may differ from other studies. Among AML cases, we found that the most common aberrant antigen was CD19, this is in contrast to the study conducted by Hamid A et al in which CD19 along with CD7 were found as common aberrant antigens in AML cases.^19^

In another study carried by El-Sissy AH et al, CD9 was the most common lymphoid antigen in AML and the most common subtype of AML in which aberrancy was reported was M3 while our study showed aberrant expressions were most commonly found in AML-M2.^20^ The variation may be reflected due to the cohorts’ age difference. The association of worse prognosis of acute leukemia with aberrant markers is shown by some studies particularly myeloid antigens expression in ALL and CD7 in AML but others have disproved such findings.^21^-^23^

### Limitations of the study:

It include use of restricted monoclonal antibodies panel for aberrant expressions’ determination due to cost restrictions and time bound sample collection. The most important missed marker is CD33 for B-ALL cases. Long term follow-up of children diagnosed with acute leukemia in terms of disease remission can provide valuable prognostic information regarding these aberrant immunophenotypic markers.

## CONCLUSION

The majority of cases with aberrant markers were seen in B-ALL (CD13) followed by AML (CD19) and then T-ALL (CD117 and HLADR). When diagnosing childhood acute leukemia, abnormal markers should be considered as they may affect the prognosis of the disease. Coexistence of more than one aberrant antigenic expressions can also be detected.

### Authors Contribution:

**NS:** Conceptualization & Contribution in manuscript writing and is responsible for the accuracy of data and maintaining integrity of work.

**SH:** Literature search, Data analysis, critical review and manuscript writing.

**FB& KM:** Literature search**,** Collection of data, Critical analysis and manuscript writing.
